# Climate and the distribution of cooperative breeding in mammals

**DOI:** 10.1098/rsos.160897

**Published:** 2017-01-18

**Authors:** Dieter Lukas, Tim Clutton-Brock

**Affiliations:** Department of Zoology, University of Cambridge, Downing Street, Cambridge CB2 3EJ, UK

**Keywords:** cooperative breeding, rainfall, sociality, phylogenetic comparison

## Abstract

Cooperative breeding systems, in which non-breeding individuals provide care for the offspring of dominant group members, occur in less than 1% of mammals and are associated with social monogamy and the production of multiple offspring per birth (polytocy). Here, we show that the distribution of alloparental care by non-breeding subordinates is associated with habitats where annual rainfall is low. A possible reason for this association is that the females of species found in arid environments are usually polytocous and this may have facilitated the evolution of alloparental care.

## 1. Introduction

**Figure 1.**
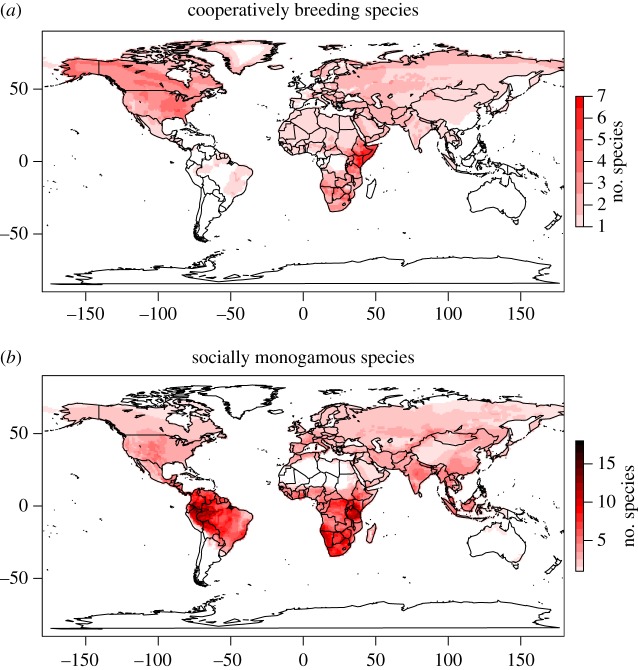
Global distribution of (*a*) cooperatively breeding and of (*b*) socially monogamous mammals. Cooperatively breeding mammals are rare, and not more than seven species can be found simultaneously in one area (*a*). Cooperative breeders tend to occur in areas that are more marginal, in contrast to most socially monogamous species in which parents receive no alloparental care which live around the equator (*b*).

Cooperative breeding systems, where non-breeding individuals care for the offspring of dominant group members, are rare among mammals, occurring primarily in rodents, carnivores and primates [1]. Phylogenetic reconstructions show that they have evolved only in lineages where females are socially monogamous (territories contain a single breeding female and male) [2] and polytocous (females produce multiple offspring per birth) [3]. Cooperative breeding also appears to be associated with arid, unpredictable habitats: a global study of breeding systems in birds has recently shown that cooperative breeding occurs in species that live in areas with low and unpredictable rainfall [4] and cooperative breeding has been shown to be associated with arid environments in mole rats [5]. Here, we compare the distribution of cooperative breeders with that of socially monogamous species (from which cooperative breeders are derived) and show that the distribution of cooperative breeding among all mammals is geographically constrained (figure 1) and associated with arid habitats.

The association between cooperative breeding and aridity raises three further questions. First, is aridity associated with the formation of breeding groups or with the provision of alloparental care or with both [6,7]? To explore this issue, we perform separate analyses of the distribution of group formation and of the presence of alloparental care. Second, is it possible that the association between cooperative breeding and aridity occurs because aridity favours the evolution of life-history parameters associated with cooperative breeding, including increases in longevity and litter size [3,8]? Third, is cooperative breeding associated with arid environments because cooperative breeders are more commonly found in arid habitats than in more mesic ones or is it a consequence of an increase in the diversity of habitats occupied by cooperative breeders? To assess whether cooperative breeders are restricted to certain environments or whether they occur in a wider range of conditions than socially monogamous species, we compare the range of climatic conditions found in areas occupied by the two groups of species, and calculate the number of different habitat types in which species in each group occur.

## 2. Material and methods

We used our previously described and published dataset on the distribution of social monogamy [9] and cooperative breeding [2]. Species were classified as socially monogamous if the majority of females are paired with a single breeding male, and as cooperative breeders if non-breeding helpers are present in most groups. We collected additional data on group formation, coding whether across the territories of socially monogamous species contain, in addition to the breeding pair, either offspring who remain in their natal group after reaching maturity or individuals joining from other groups during most of the breeding attempts. Our comparisons are restricted to terrestrial species, because socially monogamous and cooperatively breeding species are absent from the marine environments. For information about the climatic conditions in the areas where each species occurs, we extracted environmental data on annual mean in precipitation (mm) and temperature (°C), annual coefficient of variation in precipitation (variance divided by mean-squared, because rainfall cannot be smaller than zero) and variance in annual temperature, and between year predictability in precipitation and temperature from a previous review [10]. The combined dataset includes 1383 mammalian species, of which 117 are socially monogamous, as well as 32 cooperative breeders: in the primates, eight species of Cebidae (of 27); in the rodents, six species of Cricetidae (of 140), four species of Bathyergidae (of nine) and two species of Sciuridae (of 66); in the carnivores, five species of Herpestidae (of 20) and five species of Canidae (of 21) and two species of Castoridae (of two) (for full dataset, see the electronic supplementary material).

General descriptions of the main habitat types that each species occurs in were based on the IUCN classification, which characterizes habitats into 13 broad categories (desert, forest, rocky areas, caves and subterranean, savannah, grassland, shrubland, wetlands, coastal, intertidal, neritic, oceanic and artificial) [11]. Habitat diversity was calculated as the sum of habitats each species occupies. To generate figure 1, we downloaded species distribution maps from the IUCN Red List of Threatened Species [11], and plotted their combined distribution, using function of the package LetsR [12] in the statistical software R [13].

In all analyses, we included a covariance matrix reflecting shared ancestry among species, based on the updated mammalian supertree [14]. The tree was truncated to match the species in the dataset, using functions of the package ‘ape’ [15] in the statistical software R. We resolved polytomies randomly for all analyses that require bifurcating trees, and repeated each analysis with three independent resolutions, which in all cases gave consistent results. For comparisons between cooperative breeders and socially monogamous species, we performed phylogenetic ANOVA, using functions in the package ‘geiger’ in R (based on Wilks' statistic compared with null distribution based on 10 000 simulations) [16]. Binomial regressions were performed using MCMCglmm [17] in R to identify which of the climatic variables best predicts the occurrence of cooperative breeding. We included the phylogenetic relationship between species as covariance matrix, set an informative prior and used 1 000 000 iterations, a burn-in of 200 000 and a thinning interval of 10. The analysis was repeated three times, and visually inspected for convergence. Terms were considered statistically significant when the calculated pMCMC values were less than 0.05. Climatic variables are highly correlated, leading to collinearities when combining multiple variables in single models, and we assessed model stability to check whether this influences the conclusions [18]: first by comparing models including different combinations of predictor variables and second by checking whether the same climate variables that explain the occurrence of cooperative breeding are linked to either group formation or alloparental care.

## 3. Results

### 3.1. Association between the global distribution of cooperative breeding and climatic conditions

**Figure 2.**
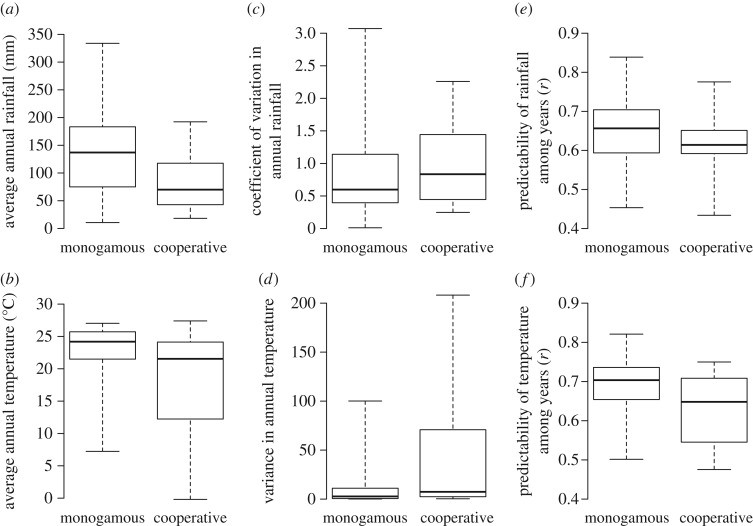
Differences in rainfall and temperature between socially monogamous and cooperative mammals. Compared with socially monogamous species in which only parents care for offspring (*n* = 117 species), cooperatively breeding species (*n* = 32 species) live in habitats that have significantly lower annual amounts of rainfall (*a*) and lower annual temperatures (*b*), significantly higher annual variation in rainfall (*c*) and temperature (*d*) and in which predictability of rainfall (*e*) and temperature (*f*) among years is significantly lower. The most distinctive climatic correlate of cooperative breeding is that cooperative breeders only occur in areas with low rainfall. In contrast, the difference in temperature between habitats occupied by cooperative breeders and those occupied by socially monogamous species that do not receive assistance from alloparents occurs because cooperative breeders occur in a wider range of temperatures. The solid line in each box shows the median value across species, the boxes contain the values of 75% of all species, and the whiskers extend to the most extreme values.

Cooperatively breeding mammals live in habitats that have significantly lower amounts of rainfall (phylogenetic ANOVA *F* = 15.7, *p* < 0.01; sample sizes for all comparisons: 117 socially monogamous species versus 32 cooperative breeders), significantly higher coefficients of variation in annual rainfall (phylogenetic ANOVA *F* = 12.03, *p* = 0.02) and significantly lower between year predictability in rainfall (phylogenetic ANOVA *F* = 5.74, *p* < 0.01) than socially monogamous species where parents are not assisted by alloparents (figure 2). The habitats of cooperatively breeding mammals also differ with regards to temperature: compared with socially monogamous species without helpers, cooperative breeders live in habitats with significantly lower annual mean temperatures (phylogenetic ANOVA *F* = 19.77, *p* < 0.01), significantly higher variance in annual temperature (phylogenetic ANOVA *F* = 19.74, *p* < 0.01) and significantly lower between year predictability in temperature (phylogenetic ANOVA *F* = 18.66, *p* < 0.01; table 1).

**Table 1.** Differences in rainfall and temperature between environments of solitary, pair-living, family-living, cooperatively breeding and group-living mammals. Compared with all socially monogamous species and socially monogamous species in which ranges contain additional individuals who do not provide alloparental care, cooperative breeders live in areas with significantly lower annual mean, annual variation and between year predictability in temperature and rainfall (values are median of the species with a respective social system; annual variation in rainfall is shown as the coefficient of variation). The climatic variables that particularly differentiate cooperative breeders from all remaining mammals are their significantly lower annual amount of rainfall.

	temperature			rainfall		
	annual mean (°C)	annual variation	between year predictability	annual mean (mm)	annual variation	between year predictability
all non-cooperative (*n* = 1351 species)	21.4	9.1	0.65	88.7	0.79	0.62
all socially monogamous (*n* = 117 species)	24.2	2.7	0.70	136.9	0.60	0.66
additional group members do not help (*n* = 19 species)	25.6	0.5	0.73	184.2	0.49	0.69
cooperative breeders (*n* = 32 species)	21.7	7.4	0.65	70.0	0.84	0.62

In multilevel analyses, annual rainfall remains the only significant climatic predictor of the distribution of cooperative breeding (table 2). Because covariances among variables mean that the full model with all six variables does not converge, we ran two models: first assessing effect of means and coefficients in annual conditions (amount of rainfall post-mean −1.76 [95% CI −3.27 to −0.26], *p* = 0.02; annual temperature post-mean −9.15 [95% CI −23.18 to 4.19], *p* = 0.17; coefficient of variation in annual rainfall post-mean −55.95 [95% CI −185.06 to 67.21], *p* = 0.37; variance in annual temperature post-mean 0.04 [95% CI −0.07 to 0.15], *p* = 0.47), and second assessing means and predictability (amount of rainfall post-mean −1.10 [95% CI −2.38 to 0.02], *p* = 0.04; annual temperature post-mean −8.81 [95% CI −26.92 to 8.07], *p* = 0.31; predictability of rainfall post-mean −444.85 [95% CI −1525.54 to 475.56], *p* = 0.32; predictability of temperature post-mean −473.67 [95% CI −1986.80 to 980.84], *p* = 0.54). In combination, average annual rainfall and social monogamy predict the occurrence of cooperative breeding across mammals (social monogamy post-mean 43.12 [95% CI 18.83–61.14], *p* < 0.001; annual rainfall post-mean −0.05 [95% CI −0.13 to −0.01], *p* = 0.003; sample sizes for all comparisons: 1351 non- versus 32 cooperative breeders). However, because arid habitats are also occupied by non-monogamous mammals that range widely, including many ungulates, average climatic conditions do not differ significantly between cooperative breeders and all other mammals (table 1; average amounts of rainfall: phylogenetic ANOVA *F* = 1.3, *p* = 0.56; coefficient of variation in annual rainfall: phylogenetic ANOVA *F* = 0.4, *p* = 0.73; between year predictability in rainfall: phylogenetic ANOVA *F* = 0.1, *p* = 0.91; average temperature: phylogenetic ANOVA *F* = 0.2, *p* = 0.84; variance in annual temperature: phylogenetic ANOVA *F* = 0.0, *p* = 0.99; between year predictability in temperature: phylogenetic ANOVA *F* = 1.7, *p* = 0.47).

**Table 2.** Annual amounts of rainfall remain the only significant factor associated with the distribution of cooperative breeding in multilevel analyses. Multilevel analyses of the climatic correlates of the distribution of cooperative breeding in mammals indicate that this breeding system is primarily associated with arid conditions, rather than with temperature or with within-year variation in climate (*a*) or with the predictability of climatic conditions among years (*b*).

		95% confidence interval		
	mean effect	lower	upper	*p*-value
(*a*)				
*annual amount of rainfall*	*−1*.*76*	*−3*.*27*	*−0*.*26*	*0*.*02*
annual variation in rainfall	−55.95	−185.06	67.21	0.37
annual average temperature	−9.15	−23.18	4.19	0.17
annual variation in temperature	0.04	−0.07	0.15	0.47
(*b*)				
*annual amount of rainfall*	*−1*.*1*	*−2*.*38*	*0*.*02*	*0*.*04*
among year predictability of rainfall	−444.85	−1525.54	475.56	0.32
annual average temperature	−8.81	−26.92	8.07	0.31
among year predictability of temperature	−473.67	−1986.8	980.84	0.54

### 3.2. Group formation, alloparental care and rainfall

None of the climatic variables we examined (annual mean, annual variation and predictability across years in rainfall or temperature) predicted whether or not socially monogamous breeding groups contain additional non-breeding adults (all phylogenetic ANOVA *p* > 0.13; sample sizes for all comparisons: 58 species in which territories only contain a single breeding pair and offspring who have not reached maturity; 51 species in which territories contain, in addition to the dominant breeders, offspring who have not dispersed despite reaching maturity or individuals who immigrated). Climatic conditions are also not associated with the number of individuals besides the breeding pair that live in a territory (annual amount of rainfall post-mean 0.006, *p* = 0.43). In contrast, differences in climate predict whether or not non-breeding group members are involved in alloparental care in species where groups include non-breeding subordinates. Species where resident non-breeders contribute to offspring care (cooperative breeders) occur in habitats with significantly lower annual amounts of rainfall than species where groups contain non-breeding adults that do not contribute to alloparental care (the only climate variable remaining significantly associated with cooperative breeding after controlling for phylogeny; phylogenetic ANOVA *F* = 70.6, *p* < 0.01; 32 cooperative breeders compared with 19 species in which additional group members do not help; multilevel analysis: amount of rainfall post-mean −2.30 [95% CI −4.44 to −0.15], *p* < 0.001; group-size post-mean 13.25 [95% CI −6.00 to 15.14], *p* = 0.26).

### 3.3. Cooperative breeding, polytocy and rainfall

Arid environments are also associated with the distribution of polytocy. In all of the 27 species in our sample living in areas with less than 50 mm of rainfall per year, females produce multiple offspring whereas, in 30 of the 49 species living in areas with more than 150 mm of rain, females are monotocous and produce single offspring (total: females are monotocous in 39 species and polytocous in 92 species). In multilevel models comparing socially monogamous and cooperatively breeding mammals, polytocy remains a significant predictor of the occurrence of cooperative breeding while variation in rainfall does not explain additional variation, suggesting that the association between rainfall and cooperative breeding may be a consequence of the association between (low) rainfall and polytocy (annual rainfall post-mean −0.09 [95% CI −0.25 to 0.05], *p* = 0.18; litter size post-mean 70.72 [95% CI 23.64–120.75], *p* = 0.002; *n* = 104 species, of which 28 are cooperative breeders).

### 3.4. Comparisons of the range of conditions experienced by cooperative breeders and socially monogamous species

**Figure 3.**
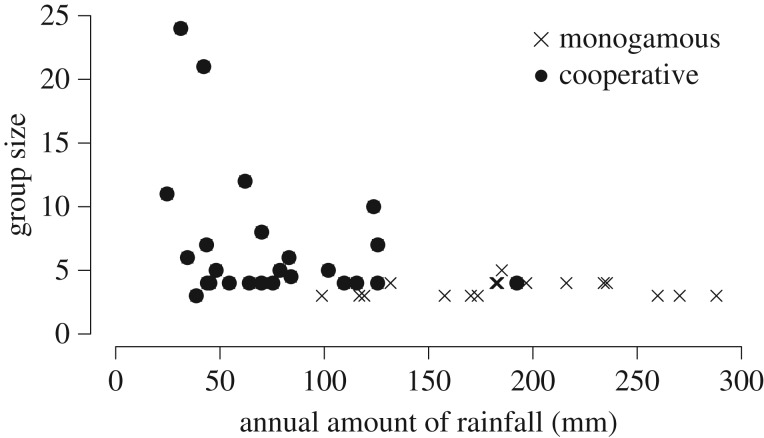
Subordinates provide cooperative care in species living in environments with low rainfall. Across socially monogamous species in which groups contain non-breeding adults, alloparental care occurs in species living in habitats with low annual amounts of rainfall, whereas helping is absent in almost all socially monogamous species in which groups contain non-breeding adults living in areas with at least 100 mm of rainfall per year (the exception are emperor tamarin monkeys). Differences in rainfall do not significantly explain variation in the number of subordinates associated with the breeding pair.

All cooperative breeders in our sample live in areas with less than 200 mm of rainfall per year, and most in areas with less than 100 mm per year (figure 2) and almost all group-forming socially monogamous species that occur in areas with less than 130 mm of rainfall per year are cooperative breeders (figure 3). In contrast, only a few cooperative breeders live in areas with more than 150 mm of rainfall per year (figure 3), and these are mainly cooperatively breeding callitrichid primates, which occur in drier areas than the other non-cooperative callitrichid species. In contrast to differences in rainfall, differences in mean and predictability of temperature between socially monogamous species and cooperative breeders occur because cooperative breeders are found in a wider range of temperatures (figure 2).

The wider range of climatic conditions where cooperative breeders occur is also evident in analyses of the diversity of habitats species occupy: cooperatively breeding mammals are found on average in three of the 13 different habitat types used to categorize global environments by the IUCN, significantly more than either socially monogamous mammals in which parents receive no help (average of two habitats; *F* = 17.1, *p* < 0.001, phylogenetic ANOVA *p* = 0.004, *n* = 111 species), species in which non-breeding group members do not provide alloparental care (average of two habitats) or all remaining mammals (average of two habitats; *F* = 8.7, *p* = 0.003, phylogenetic ANOVA *p* = 0.15). The difference with socially monogamous species is not simply owing to cooperative breeding occurring in lineages that occupy more diverse habitats: as indicated by the analysis correcting for phylogenetic similarity, cooperative breeders appear to consistently live in more habitat types (e.g. cooperatively breeding carnivores live on average in 4.4 different habitat types while socially monogamous carnivores live in 3.6; cooperatively breeding primates live on average in 1.4 different habitat types, whereas socially monogamous primates live in 1.2). In particular, cooperatively breeding species are more likely to occur in open rather than in closed habitats, and may be better able to live in artificial habitats (table 3).

**Table 3.** Proportion of socially monogamous and of cooperatively breeding mammals occupying certain habitats. Habitat definitions are taken from the IUCN red list. Proportions reflect the number of either cooperatively breeding (*n* = 32) or socially monogamous (*n* = 159) species found in different habitats. Species can occupy more than one habitat, with cooperatively breeding species found on average in three and socially monogamous species on average in two different habitats. Cooperatively breeding species are more likely to occur in open rather than closed habitats, and appear better able to live in artificial habitats.

	socially monogamous (%)	cooperative breeder (%)
forest	78	59
desert	6	16
savannah	21	44
shrublands	27	50
grasslands	22	50
wetlands	7	22
rocky	8	13
intertidal	2	6
coastal	1	3
caves	3	3
neritic	0	0
oceanic	0	0
artificial	22	41

## 4. Discussion

Our results suggest that mammals that breed cooperatively occur in more and different environments than those occupied by socially monogamous species. Aridity is associated with whether additional group members provide alloparental care, whereas the presence of resident non-breeding subordinates appears not to be associated with climate differences. Our study indicates that the range of temperatures and habitat categories occupied by cooperative breeders is relatively wide. However, this still leaves the possibility that small differences in rainfall might have favoured the initial transition to cooperative breeding, followed by subsequent spread into larger areas.

A potential explanation for the association between alloparental care and low rainfall is that low average resource availability in arid environments has led to reproductive suppression and alloparental care: if females without helpers face high risks of reproductive failure in arid environments [19], a subordinate's best strategy might be to queue within an established group and to contribute to territory defence [20] and to alloparental care, ensuring they start breeding in a group with stable access to food and with many helpers [7,21]. Alternatively (or additionally) arid environments where resource availability fluctuates widely may lead to selection for increased fecundity to maximize reproduction in years when breeding is possible as well as to selection on non-breeders to provide assistance in rearing young [22,23]. Selection for alloparental care could have been reinforced by the association between arid environments and polytocy, because the production of litters is usually associated both with increases in the energetic costs of breeding and with higher coefficients of relatedness between group members [24].

The differences in annual means and predictability of rainfall and temperature between the environments occupied by pair-living and cooperatively breeding mammals show some similarities with the patterns previously described in birds, in which almost all non-cooperative species live in pairs [3]. Both in birds and in mammals cooperatively breeding species are found in areas with low rainfall that differ in predictability of climate from areas in which pair-living species are found. However, cooperatively breeding birds and mammals appear to occur in different temperature niches (birds: warm versus mammals: cold), and it is as yet unclear whether, as in mammals, low rainfall is specifically associated with helping behaviour in birds. Differences in life history between the clades might modify how environmental factors influence the evolution of cooperative breeding [5]. For example, most birds breed seasonally, and longevity is associated with group formation in birds [25,26]. In contrast, cooperatively breeding mammals tend to have multiple breeding attempts per year [7], and reproductive rate rather than longevity might be a more important contributor to variance in lifetime reproductive success.

Our results show that aridity predicts the global distribution of cooperative breeding in mammals, and that it is one of multiple factors influencing the occurrence of cooperative breeding systems. In addition to kinship, ecological and life-history factors interact to influence costs and benefits for both dominants and helpers [5,7]. Further insights into these interactions are likely to come from interpopulation comparisons [19,27,28], which offer opportunities to investigate the effects of helpers on the growth and survival of juveniles as well as on the fecundity and survival of breeders under contrasting ecological conditions.

## Supplementary Material

Lukas&CluttonBrock_SupplementaryData_ClimateCoopBreeding.csv

## Data accessibility

All data are available in the supplementary material and at the KNB Data Repository: https://knb.ecoinformatics.org.

## References

[1] Solomon NG, French JA (eds). 1997 Cooperative breeding in mammals. Cambridge, UK: Cambridge University Press.

[2] Lukas D, Clutton-Brock T 2012 Cooperative breeding and monogamy in mammalian societies. Proc. R. Soc. B 279, 2151–2156. (doi:10.1098/rspb.2011.2468) 10.1098/rspb.2011.2468 PMC3321711 22279167

[3] Lukas D, Clutton-Brock T 2012 Life histories and the evolution of cooperative breeding in mammals. Proc. R. Soc. B 297, 4065–4070. (doi:10.1098/rspb.2012.1433) 10.1098/rspb.2012.1433 PMC3427589 22874752

[4] Jetz W, Rubenstein DR 2011 Environmental uncertainty and the global biogeography of cooperative breeding in birds. Curr. Biol. 21, 72–78. (doi:10.1016/j.cub.2010.11.075) 21185192 10.1016/j.cub.2010.11.075

[5] Faulkes CG, Bennett NC, Bruford MW, O'Brien HP, Aguilar GH, Jarvis JU 1997 Ecological constraints drive social evolution in the African mole–rats. Proc. R. Soc. Lond. B 264, 1619–1627. (doi:10.1098/rspb.1997.0226) 10.1098/rspb.1997.0226 PMC1688729 9404025

[6] Hatchwell BJ, Komdeur J 2000 Ecological constraints, life history traits and the evolution of cooperative breeding. Anim. Behav. 59, 1079–1086. (doi:10.1006/anbe.2000.1394) 10877885 10.1006/anbe.2000.1394

[7] Clutton-Brock T 2002 Breeding together: kin selection and mutualism in cooperative vertebrates. Science 296, 69–72. (doi:10.1126/science.296.5565.69) 11935014 10.1126/science.296.5565.69

[8] Tökölyi J, Schmidt J, Barta Z 2014 Climate and mammalian life histories. Biol. J. Linn. Soc. 111, 719–736. (doi:10.1111/bij.12238)

[9] Lukas D, Clutton-Brock TH 2013 The evolution of social monogamy in mammals. Science 341, 526–530. (doi:10.1126/science.1238677) 23896459 10.1126/science.1238677

[10] Botero CA, Dor R, McCain CM, Safran RJ 2014 Environmental harshness is positively correlated with intraspecific divergence in mammals and birds. Mol. Ecol. 23, 259–268. (doi:10.1111/mec.12572) 24283535 10.1111/mec.12572

[11] IUCN. 2014 The IUCN Red List of Threatened Species. Version 2014.1. http://iucnredlist.org (accessed 12 June 2015).

[12] Vilela B, Villalobos F 2015 letsR: a new R package for data handling and analysis in macroecology. Methods Ecol. Evol. 6, 1229–1234. (doi:10.1111/2041-210X.12401)

[13] R Development Core Team. 2010 R: a language and environment for statistical computing. Vienna, Austria: R Foundation for Statistical Computing.

[14] Rolland J, Condamine FL, Jiguet F, Morlon H 2014 Faster speciation and reduced extinction in the tropics contribute to the mammalian latitudinal diversity gradient. PLoS Biol. 28, e1001775 (doi:10.1371/journal.pbio.1001775) 10.1371/journal.pbio.1001775 PMC3904837 24492316

[15] Paradis E, Claude J, Strimmer K 2004 APE: analyses of phylogenetics and evolution in R language. Bioinformatics 20, 289–290. (doi:10.1093/bioinformatics/btg412) 14734327 10.1093/bioinformatics/btg412

[16] Harmon LJ, Weir JT, Brock CD, Glor RE, Challenger W 2008 GEIGER: investigating evolutionary radiations. Bioinformatics 24, 129–131. (doi:10.1093/bioinformatics/btm538) 18006550 10.1093/bioinformatics/btm538

[17] Hadfield JD 2010 MCMC methods for multi-response generalized linear mixed models: the MCMCglmm R package. J. Stat. Softw. 33, 1–22. (doi:10.18637/jss.v033.i02)20808728

[18] Mundry R 2014 Statistical issues and assumptions of phylogenetic generalized least squares. In Modern phylogenetic comparative methods and their application in evolutionary biology (ed. Garamszegi L), pp. 131–153. Berlin, Germany: Springer.

[19] Rubenstein DR 2011 Spatiotemporal environmental variation, risk aversion, and the evolution of cooperative breeding as a bet-hedging strategy. Proc. Natl Acad. Sci. USA 108, 10 816–10 822. (doi:10.1073/pnas.1100303108) 10.1073/pnas.1100303108 PMC3131817 21690415

[20] Gaston AJ 1978 The evolution of group territorial behavior and cooperative breeding. Am. Nat. 112, 1091–1100. (doi:10.1086/283348)

[21] Stacey PB, Ligon JD 1991 The benefits-of-philopatry hypothesis for the evolution of cooperative breeding: variation in territory quality and group size effects. Am. Nat. 137, 831–846. (doi:10.1086/285196)

[22] Reyer HU 1984 Investment and relatedness: a cost/benefit analysis of breeding and helping in the pied kingfisher (*Ceryle rudis*). Anim. Behav. 32, 1163–1178. (doi:10.1016/S0003-3472(84)80233-X)

[23] Creel SR, Creel NM 1991 Energetics, reproductive suppression and obligate communal breeding in carnivores. Behav. Ecol. Sociobiol. 28, 263–270. (doi:10.1007/BF00175099)

[24] Beckerman AP, Sharp SP, Hatchwell BJ 2011 Predation and kin-structured populations: an empirical perspective on the evolution of cooperation. Behav. Ecol. 22, 1294–1303. (doi:10.1093/beheco/arr131)

[25] Arnold KE, Owens IP 1998 Cooperative breeding in birds: a comparative test of the life history hypothesis. Proc. R. Soc. Lond. B 265, 739–745. (doi:10.1098/rspb.1998.0355)

[26] Downing PA, Cornwallis CK, Griffin AS 2015 Sex, long life and the evolutionary transition to cooperative breeding in birds. Proc. R. Soc. B 282, 20151663 (doi:10.1098/rspb.2015.1663) 10.1098/rspb.2015.1663 PMC4614776 26400743

[27] Bateman AW, Ozgul A, Nielsen JF, Coulson T, Clutton-Brock TH 2013 Social structure mediates environmental effects on group size in an obligate cooperative breeder, *Suricata suricatta*. Ecology 94, 587–597. (doi:10.1890/11-2122.1) 23687885 10.1890/11-2122.1

[28] Sheehan MJ, Botero CA, Hendry TA, Sedio BE, Jandt JM, Weiner S, Toth AL, Tibbetts EA 2015 Different axes of environmental variation explain the presence vs. extent of cooperative nest founding associations in *Polistes* paper wasps. Ecol. Lett. 18, 1057–1067. (doi:10.1111/ele.12488) 26248800 10.1111/ele.12488 PMC4564336

